# Multispecies Probiotic Supplementation Favorably Affects Vascular Function and Reduces Arterial Stiffness in Obese Postmenopausal Women—A 12-Week Placebo-Controlled and Randomized Clinical Study

**DOI:** 10.3390/nu10111672

**Published:** 2018-11-05

**Authors:** Monika Szulińska, Igor Łoniewski, Katarzyna Skrypnik, Magdalena Sobieska, Katarzyna Korybalska, Joanna Suliburska, Paweł Bogdański

**Affiliations:** 1 Department of Treatment of Obesity, Metabolic Disorders and Clinical Dietetics, University of Medical Sciences in Poznań, Szamarzewskiego Str. 84, 60-569 Poznań, Poland; mszulinska1@wp.pl (M.S.); pawelbogdanski73@gmail.com (P.B.); 2 Department of Biochemistry and Human Nutrition, Pomeranian Medical University in Szczecin, Broniewskiego 24, 71-460 Szczecin, Poland; 3 Institute of Human Nutrition and Dietetics, Poznan University of Life Sciences, Wojska Polskiego St. 31, 60-624 Poznań, Poland; katarzyna.skrypnik@gmail.com (K.S.); jsulibur@up.poznan.pl (J.S.); 4 Department of Rheumatology and Rehabilitation, Poznan University of Medical Sciences, 28. Czerwca 1956r 135/147, 61-55 Poznań, Poland; msobieska@ump.edu.pl; 5 Department of Pathophysiology, Poznan University of Medical Sciences, Rokietnicka 8, 60-806 Poznan, Poland; koryb@ump.edu.pl

**Keywords:** probiotics, endothelium, arterial stiffness, postmenopausal women

## Abstract

Obesity in the postmenopausal period is associated with an increased risk of cardiovascular diseases in women. One of the key drivers of cardiovascular risk is endothelial dysfunction; thus, this is also a crucial point for studies on new therapeutic methods of cardioprotective properties. The aim of the current study was to evaluate the effect of two doses of multispecies probiotic Ecologic^®^ Barrier supplement on functional (primary endpoint) and biochemical parameters (secondary endpoint) of endothelial dysfunction in obese postmenopausal women in a 12-week randomized, placebo-controlled clinical trial. A total of 81 obese Caucasian women participated in the trial. The subjects were randomly assigned to three groups that received a placebo, a low dose (LD) (2.5 × 10^9^ colony forming units (CFU) per day), or a high dose (HD) (1 × 10^10^ CFU per day) of lyophilisate powder containing live multispecies probiotic bacteria. The probiotic supplement was administered each day for 12 weeks in two equal portions. A high dose probiotic supplementation for 12 weeks decreased systolic blood pressure, vascular endothelial growth factor, pulse wave analysis systolic pressure, pulse wave analysis pulse pressure, pulse wave analysis augmentation index, pulse wave velocity, interleukin-6, tumor necrosis factor alpha, and thrombomodulin. Low doses of probiotic supplementation decreased the systolic blood pressure and interleukin-6 levels. The mean changes in the estimated parameters, compared among the three groups, revealed significant differences in the vascular endothelial growth factor, the pulse wave analysis systolic pressure, the pulse wave analysis augmentation index, the pulse wave velocity, the tumor necrosis factor alpha, and thrombomodulin. The post hoc tests showed significant differences for all parameters between HD and the placebo group, and HD and LD (besides pulse wave analysis augmentation index). We show for the first time that supplementation with multispecies probiotic Ecologic^®^ Barrier favorably modifies both functional and biochemical markers of vascular dysfunction in obese postmenopausal women.

## 1. Introduction

Increased scientific interest in the endothelium has risen from the fact that beyond any doubt, an unfavorable modification of endothelial cells, leading to endothelial dysfunction, is closely associated with an increased risk for cardiovascular diseases [1]. Changes associated with the deterioration of endothelial function are responsible for vascular remodeling and the activation of inflammatory and thrombotic processes, and they are early markers of atherosclerosis development [2]. Detailed knowledge about pathologic mechanisms leading to endothelial dysfunction in obesity is a crucial point for studies on new therapeutic methods of cardioprotective properties.

Obesity, a global epidemic affecting 700 million people worldwide, disrupts endothelial function. In obese patients, enhanced vasoconstriction is observed, mainly due to endothelincyclooxygenase hyperactivity [3], and angiotensin-1 receptor hyper-production occurs, which could be the basis of hypertension in these individuals [4]. It has been widely documented that visceral obesity significantly aggravates endothelial dysfunction [5,6]. Obesity promotes a low-intensity inflammatory state, leading to the over-secretion of inflammation mediators such as the tumor necrosis factor (TNF) and interleukin-6 (IL-6), which significantly affect endothelium function [7] and homeostasis. The regulation of angiogenesis by endothelial cells is carried out by the expression of receptor proteins on their surface, and the synthesis of numerous proangiogenic factors, among which the most important seems to be the vascular endothelial growth factor (VEGF), as well as anti-angiogenesis factors (protease inhibitors: tissue inhibitors of metalloproteinases (TIMPs), thrombospondin 1 and 2) [8]. These processes are disturbed in obesity. The proangiogenic activity of VEGF is multidirectional, and it includes chemotactic and mitogenic effects, an increase in the migration of endothelial cells and their progenitor cells, and activity-degrading basement membranes, which lead to an increased blood vessel permeability [9]. Simultaneously, the endothelium influences fibrinolysis-producing tissue plasminogen activator and plasminogen activator inhibitor 1, and can be a source of the von Willebrand factor (vWF) and thrombomodulin (TM)—important coagulation factors [10,11,12]. TM interacts with the thrombin that is present in the plasma and contributes to changes in its conformation. In obesity, due to the ability of TM to be cleaved by inflammatory agents, it can be considered a marker of endothelial cell damage [13]. Endothelial cells are a source of vWF and play an important role in the process of platelet adhesion and aggregation [14].

An understanding of the importance of ED in the pathology of cardiovascular diseases has led to the development of methods to measure the grade of this dysfunction. Commonly used methods of ED examination are biochemical measurements and non-invasive functional tests. The basic method of endothelium examination based on applanation tonometry allows for the evaluation of pulse wave velocity (PWV). The value of the PWV index is inversely proportional to the stiffness of the arterial wall. The predictive value of this method grows rapidly [15,16].

An understanding of the importance of endothelial dysfunction in the pathology of cardiovascular diseases allows for the capabilities of endothelial function to be investigated and modified. Non-pharmacological interventions in endothelial function are widely available, cheap, devoid of side effects, and attractive. An increasing amount of scientific proof confirms its favorable cardioprotective impact; thus, further studies on this topic are needful. In recent years, the field of non-pharmacological interventions has become of special interest for our research team [17,18,19,20,21]. Probiotics are considered to be alternative supplements that can modulate the composition and function of gut microbiota. An improper diet and a sedentary lifestyle lead to obesity, which has effects on chronic inflammatory responses. Sub-clinical inflammation is a reason for dysbiosis and the increased permeability of the intestinal wall. Due to this phenomenon, bacterial lipopolysaccharides penetrate into the bloodstream, and cause an increased production of trimethylamine N-oxide (TMAO) and decreased TAS (total antioxidant status). The entirety of these processes leads to endothelial dysfunction [22]. The strain-specific capacities of bacteria in the multispecies probiotic Eccologic^®^ Barrier that are designed to improve the epithelial barrier have been recently investigated [23]. The administration of the Ecologic^®^ Barrier was also shown to improve insulin resistance and reduced abdominal adiposity in type 2 diabetes mellitus (T2DM) patients [24]. In our recently published 12-week randomized, placebo-controlled, double-blind study, we showed that supplementation with Ecologic^®^ Barrier in obese postmenopausal women favorably influences cardiometabolic parameters and gut permeability [25]. Our results suggest that these products can be effective in the prevention and treatment of cardiovascular diseases in obese postmenopausal women. Although the multiple potential effects of probiotics have been studied, no data regarding the influence of multispecies probiotics on endothelial function in obese postmenopausal women are available so far. Women of postmenopausal age develop an increased risk of developing cardiovascular diseases due to multiple hormonal changes. Our previous results obtained in obese individuals led us to investigate the hypothesis concerning the positive effect of supplementation with the multispecies probiotic Ecologic^®^ Barrier on further endothelium-related, cardiovascular risk factors in female patients with obesity.

The aim of the study was to evaluate the effects of different doses of supplemented multispecies probiotics on the functional (primary endpoint) and biochemical parameters (secondary endpoint) of endothelial and vascular dysfunction in obese postmenopausal women in a 12-week randomized, placebo-controlled clinical trial.

## 2. Methods

The study was designed as a 12-week single-center (Department of Education and Treatment of Obesity and Metabolic Disorders University of Medical Sciences in Poznań, Poland), randomized, double-blind, placebo-controlled clinical trial. The protocol was registered at the US National Institute of Health (ClinicalTrails.gov Identifier: NCT03100162). Ethical approval was obtained from the Bioethical Committee of Poznan University of Medical Sciences (No. 871/2015) and written informed consent was obtained from all participants prior to inclusion. The study took place from 27 February 2016 to 31 December 2017.

### 2.1. Subjects

**Figure 1:**
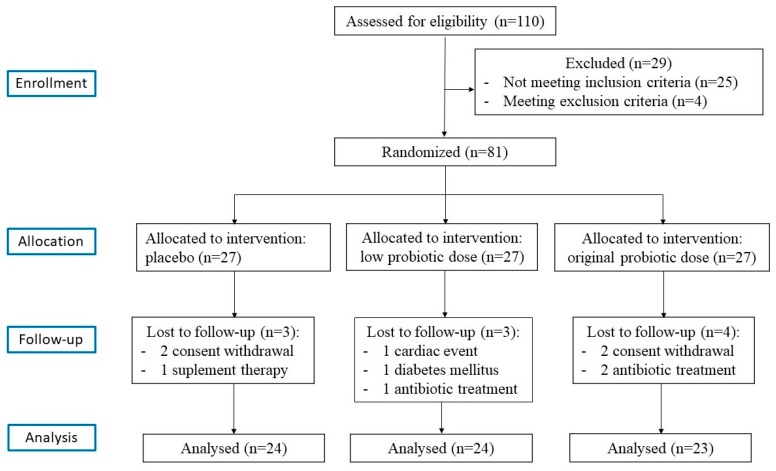
A flowchart of the study design.

In the current study, the same participant sample was used, as in the study previously published by our group [25]. All patients were recruited from the outpatient department of the University Hospital, Poznań, Poland. A total of 110 obese postmenopausal women were initially invited to participate. The inclusion criteria were as follows: (1) women aged 45–70 years; (2) ≥6 months since last menstruation; (3) a body mass index (BMI) of 30–45 kg/m^2^; (4) abdominal obesity, that is, a waist circumference of >80 cm (International Diabetes Federation 2005); (5) a content of body fat assessed by electrical bioimpedance that is ≥33%; and (6) a stable body weight in the month prior to the trial (permissible deviation ±1 kg). Patients presenting any of the following exclusion criteria were excluded from the study: (1) secondary forms of obesity; (2) gastrointestinal diseases; (3) diabetes; (4) pharmacotherapy of hypertension or dyslipidemia in the three months prior to the trial; (5) history of use of any dietary supplements within three months before the study; (6) intake of antibiotics within one month before the study; (7) clinically significant acute inflammatory processes; (8) nicotine, alcohol, or drug abuse; (9) participation in weight-management studies or the use of medications known to alter food intake or body weight; (10) vegetarian dietary habits; (11) the use of pre- and probiotic-enriched products (for at least three weeks before the screening visit of the study), and products with a high content of dietary fiber or high quantities of fermented food (>400 g/day); and (12) hormonal replacement therapy. The occurrence of any of the above exclusion criteria during the trial resulted in the immediate cessation of participation in the study. Twenty-nine patients did not qualify for the study due to improper inclusion/exclusion criteria. Finally, 81 consecutive women diagnosed with obesity were eligible and gave informed consent. They were randomly assigned to either placebo or probiotic sachets that in a double-blinded manner. Finally, 71 participants (Placebo group, *n* = 24; the low dose of the probiotic (LD) group, *n* = 24, high dose of the probiotic (HD) group, *n* = 23) completed the 12-week intervention. No serious adverse reactions were reported following the consumption of multispecies probiotic supplements in postmenopausal women with obesity throughout the study. Furthermore, patients did not take any new medicines during the study. For a number of patients, a follow-up was not possible, and the reasons for this are outlined in the flowchart (Figure 1).

### 2.2. Probiotic Supplements and Allocation

All eligible and consenting participants were given a unique code as an identifier. They were allocated (1:1:1) to receive either probiotics (high or low dose) or a placebo. The randomization scheme was computer-generated by Winclove using permuted blocks with a block size of 4. It was impossible for the research personnel involved with the participants to adjust the randomization, or to discern which product the participants were receiving, ensuring true allocation concealment. The probiotic group received sachets containing 2 g of freeze-dried powder of the probiotic mixture Ecologic^®^ Barrier (Winclove probiotics, Amsterdam, The Netherlands). The HD group received Ecologic^®^ Barrier HD (2 g sachets, 2 sachets per day, 2.5 × 10^9^ colony-forming units (CFU) per gram = 1 × 10^10^ per day), whereas the LD group received Ecologic^®^ Barrier LD (2 g sachets, 2 sachets per day, 0.625 × 10^9^ CFU/g = 2.5 × 10^9^ per day). The probiotic preparation contained the following bacterial strains: *Bifidobacterium bifidum* W23, *Bifidobacterium lactis* W51, *Bifidobacterium lactis* W52, *Lactobacillus acidophilus* W37, *Lactobacillus brevis* W63, *Lactobacillus casei* W56, *Lactobacillus salivarius* W24, *Lactococcus lactis* W19, and *Lactococcus lactis* W58 (a total cell count of 2.5 × 10^9^/g). All strains were present in approximately equal amounts, and the quality of the study batch was tested every three months to confirm the viability of the strains. With the application of new molecular identification techniques (including whole genome sequencing), the declaration of the bacterial strains for Ecologic^®^ Barrier was updated from previous publications [23]. It has been confirmed that the probiotic formulation has always contained the nine mentioned strains, and it has not been changed in ratio or CFU count since it has been (commercially) available. The placebo group received the same sachets consisting of the carrier of the probiotic product, which was maize starch and maltodextrins. The placebo was indistinguishable in color, smell, and taste from the probiotic sachets. All participants were asked to consume two sachets per day (by dissolving the contents in a glass of water), once before breakfast and once before going to bed. Participants were asked to return every four weeks to surrender unused sachets and to be given fresh refills to monitor compliance. Participants were asked not to alter their routine physical activity or their usual diet. Participants were also asked to report any side effects.

### 2.3. Anthropometric Measurement

At the baseline and after 12 weeks of treatment, the anthropometric parameters were evaluated and all laboratory tests were performed for each group. All measurements were obtained after an overnight fast. The weight was measured to the nearest 0.1 kg and height was estimated to the nearest 0.5 cm. The body mass index (BMI) was calculated as weight divided by height squared (kg/m^2^).

### 2.4. Functional Parameters

Blood pressure (BP) was measured using a brachial cuff (Omron Healthcare, Kyoto, Japan) on the right arm in a sitting position. The measurements were taken three times at 2-min intervals after a 10 min rest, and the average was used for analysis.

Both pulse wave velocity (PWV) and pulse wave analysis (PWA) measurements were then performed using the SphygmoCor Px (Atcor Medical Blood Pressure Analysis System, Sydney, Australia) in a temperature-controlled room, before making anthropometric measurements and blood collection, in the Department of Education and Treatment of Obesity and Metabolic Disorders University of Medical Sciences in Poznań, Poland. PWV was evaluated between the carotid and femoral artery, with the participant lying in the supine position. Pulse measurements were performed non-invasively using the SphygmoCor probe over the carotid and femoral artery, while an electrocardiogram (ECG) recording was performed simultaneously [26]. To ensure a stable, artifact-free ECG, the skin was properly prepared (the hair was removed at the electrode site and the skin was cleaned with an alcohol wipe). A minimum of 12 s of the signal (approximately 10 heartbeats) was recorded after a strong accurate and reproducible pulse wave signal had been obtained. The distance from the carotid to the femoral artery was measured directly between each artery location and the suprasternal notch, and the values were entered into the SphygmoCor software database. PWV was calculated by measuring the time delay between two characteristic timing points on two pressure waveforms that were at a known distance apart. The SphygmoCor method uses the foot of the waveform as an onset point for calculating the time differences between the R wave of the ECG, and the pulse waveforms at each site. The PWV was automatically calculated by the Atcor software as the carotid–femoral artery distance divided by the wave traveling time between the above two measuring sites. PWV measurements with a standard deviation of less than 10% were used for analysis [27].

The central aortic hemodynamic parameters, augmentation index (Alx), aortic pressure (AP), and pulse pressure (PP), were measured using the applanation tonometry of the radial artery, as previously described [28]. Two pressure peaks characterize the systolic part of the central waveform. The first peak results from the left cardiac ventricle ejection, and the second one results from the wave reflections from the periphery. The difference between these two peaks represents the degree of the central arterial pressure augmentation due to wave reflection. The AP is the absolute increase of the PP due to the reflected wave, and AI75 is the measure of the contribution of the wave reflection to the arterial pressure waveform. AI75 is expressed as a percentage of the PP. The amplitude and timing of the reflected wave ultimately depend on the stiffness of the small vessels and large arteries, representing a measurement of the systemic arterial stiffness [26,28].

### 2.5. Biochemical Parameters

The serum level of high sensitivity (hs) TNF-α was measured using an enzyme immunoassay (DRG Instruments GmbH, Marburg, Germany). The sensitivity of the test was 0.7 pg/mL. The concentration of hs Interleukin (IL)-6 was determined in the serum samples with an immunoassay method (ELISA). For this purpose, an EIA-4640 kit, Interleukin 6 Human ELISA Kit (DRG Instruments GmbH, Marburg, Germany) was used. The sensitivity of the test was 2.0 pg/mL.

The serum level of VEGF was measured using a quantitative immunoassay (R&D Systems, Minneapolis, MN, USA). The serum thrombomodulin (TM) concentration was determined by an immunoenzymatic ELISA using the IMUBIND Thrombomodulin ELISA Kit (American Diagnostica Inc., Stamford, CT, USA). A commercially available ELISA kit (R&D Systems, Minneapolis, MN, USA) was used for the determination of levels of the von Willebrand factor.

### 2.6. In Vitro Assay

Cell culture studies were performed using human umbilical vein endothelial cells HUVEC EA.hy926 line (kindly donated by Dr. C.J.S. Edgell, University of North Carolina, Chapel Hill, NC, USA) [29]. All cell culture reagents were from Sigma (St. Louis, MI, USA) and the cell culture plastics were from Nunc (Roskilde, Denmark). Cells were routinely maintained in Earle’s buffered M199 culture medium supplemented with l-glutamine (2 mM), penicillin (100 U/mL), streptomycin (100 µg/mL), hydrocortisone (0.4 µg/mL), and 10% (*v*/*v*) of foetal calf serum (Invitrogen, Karlsruhe, Germany). For the experiments, the cells were seeded into multi-well plates at a density of 4000 cells/cm^2^ and allowed to adhere for 24 h. After that, the cells were rendered quiescent by serum deprivation for 24 h and exposed in triplicate to 20% (*v*/*v*) patients’ serum (placebo, HD, LD groups) for the next 24 h. Cells were then assessed for growth using the MTT (Methyl-Tetrazolium-Test) conversion assay [30], as previously described [31]. Briefly, following a 24-h exposure to medium supplemented with patients’ serum, cells were incubated for 4 h at 37 °C with 1.25 mg/mL MTT salt (3-(4,5-dimethylthiazol-2-yl) -2,5-diphenyl-tetrazolium bromide). The formazan product generated was dissolved with an acidic solution of 20% (*w*/*v*) sodium dodecyl sulfate and 50% (*v*/*v*) *N*,*N*-dimethylformamide. The absorbance of the converted dye was recorded at 595 nm. The data was expressed as a percentage of the control (cells maintained in a normal culture medium). The measurement was performed separately for each subject.

### 2.7. Statistical Analysis

The subjects’ randomization codes were concealed until the statistical analysis. The data are shown as means ± standard deviations (SDs). The normal distribution for each group was checked by the Shapiro–Wilk test. To examine the differences among groups, the Kruskal–Wallis test with a post-test (a multiple comparison test) or a one-way analysis of variance (ANOVA) test with a post-test (Tukey test) was used (if the data was normally distributed). To determine the differences between the effects of the treatment, the Wilcoxon test or the paired *t*-test was conducted (if the data was normally distributed). The standardized mean differences (Cohen’s d) were used as a magnitude of the effect. Effect size thresholds of 0.2, 0.5, and 0.8 were used for small, medium, and large effects, respectively. Statistics were performed with the STATISTICA (data analysis software system), version 12 (StatSoft, Inc., Tulsa, OK, USA). A *p* value of <0.05 was regarded as significant.

## 3. Results

The baseline characteristics of the studied population are shown in Table 1. There were no statistically significant differences in analyzed parameters among the HD, LD, and placebo groups at the baseline, except for VEGF (Table 1). At the baseline, the serum levels of VEGF were higher (*p* < 0.001) in the HD group in comparison to the placebo group.

**Figure 2:**
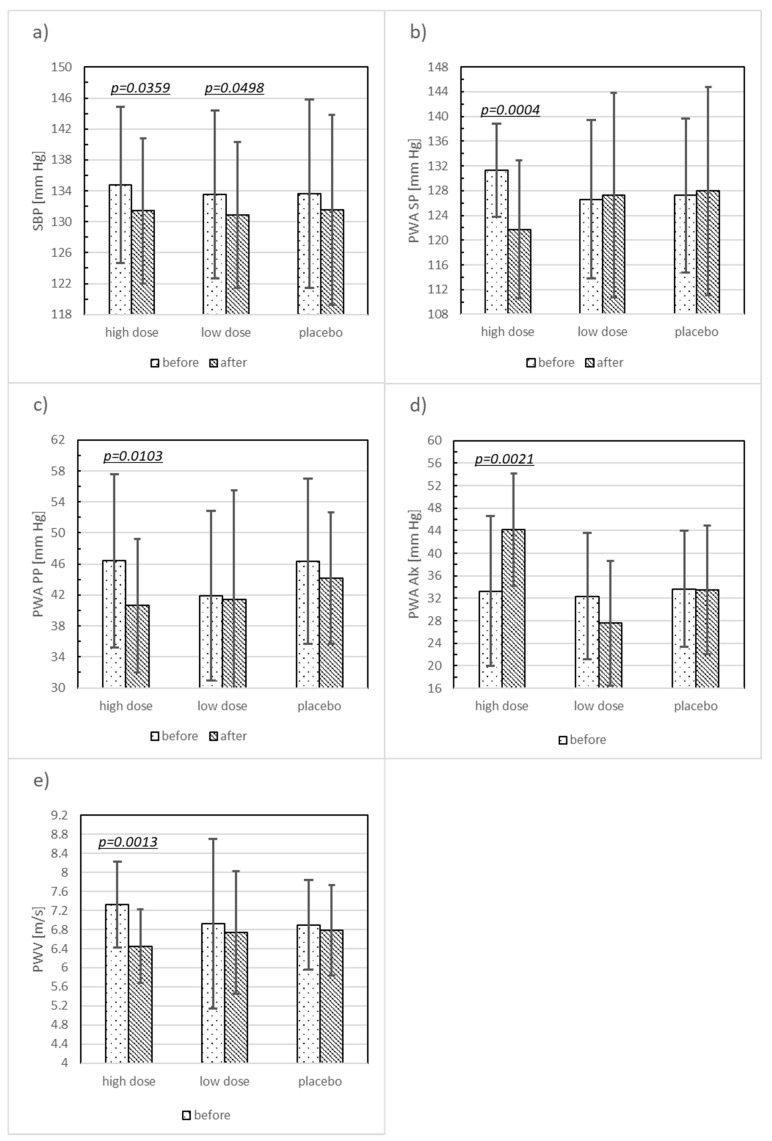
The comparison of the functional parameters in the high dose (HD), low dose (LD), and placebo groups at the beginning of the study and after three months of probiotic supplementation. Significant differences are highlighted showing the *p*-value. Data are the arithmetic mean ± SD; (**a**) SBP: systolic blood pressure; (**b**) PWA SP: pulse wave analysis systolic pressure; (**c**) PWV: pulse wave velocity; (**d**) PWA Alx: pulse wave analysis augmentation index; (**e**) PVV: pulse wave velocity.

**Figure 3:**
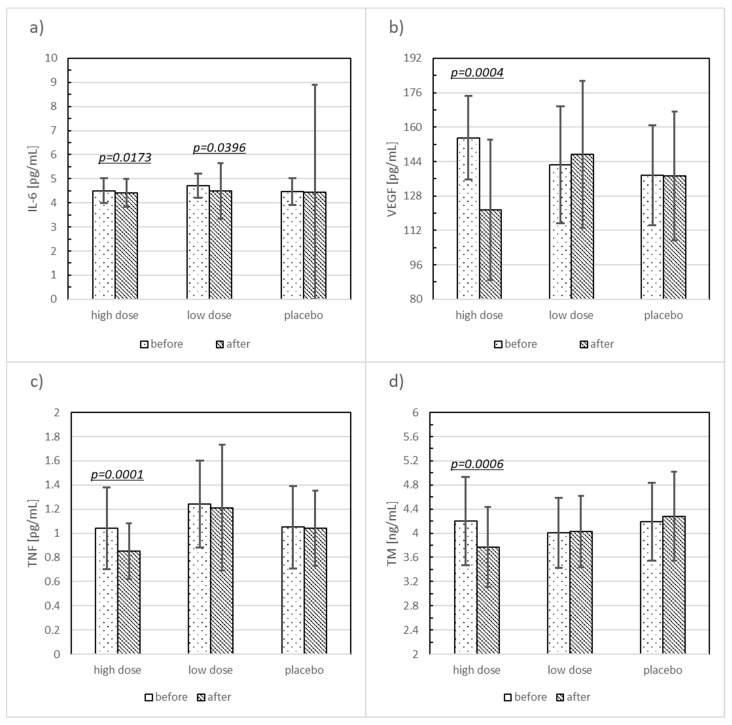
The comparison of the biochemical parameters in the high dose (HD), low dose (LD), and placebo groups at the beginning of the study and after three months of probiotic supplementation. Significant differences are highlighted showing the *p*-value. Data are arithmetic mean ± SD; (**a**) Il-6: interleukin-6; (**b**) VEGF: vascular endothelial growth factor; (**c**) TNF: tumor necrosis factor alpha; (**d**) TM: thrombomodulin.

Significant changes in some evaluated parameters, as compared before and after 12 weeks of supplementation, were found in both the HD and LD probiotic supplemented groups, but not in the placebo group (Table 2, Figure 2 and Figure 3). High doses of probiotic supplementation for 12 weeks decreased several parameters: SBP by 2.52% (*p* < 0.0359, SMD = 0.39), VEGF by 21.62% (*p* < 0.0004, SMD = 1.73), PWA SP by 7.29% (*p* < 0.0004, SMD = 1.01), PWA PP by 12.51% (*p* < 0.0103, SMD = 0.67), PWA Alx by 17.18% (*p* < 0.0021, SMD = 0.58), PWV by 11.89% (*p* < 0.0013, SMD = 1.13), Il-6 by 2% (*p* < 0.0173, SMD = 0.15), TNF-α by 18.27% (*p* < 0.0001, SMD = 0.56), and TM by 10.24% (*p* < 0.0006, SMD = 0.59). Low doses of probiotic supplementation decreased SBP by 1.96% (*p* < 0.0498, SMD = 0.26) and Il-6 by 4.46% (*p* < 0.0396, SMD = 0.43).

The mean Δ of the estimated parameters compared among the three groups revealed significant differences in VEGF (*p* < 0.0009), PWA SP (0.0016), PWA Alx (0.0084), PWV (*p* < 0.0028), TNF (*p* < 0.0012), and TM (*p* < 0.0172).

The post hoc test showed statistically significant ΔVEGF (*p* < 0.0001, SMD = −1.03), ΔPWA SP (*p* = 0.0054, SMD = −1.00), ΔPWA Alx (*p* = 0.0079, SMD = −0.55), ΔPWV (*p* = 0.0045, SMD = −0.82), ΔTNF (*p* < 0.0009, SMD = −1.03), ΔTM (*p* < 0.0194, SMD = −0.78) when comparing the HD to the placebo group. For the HD and LD group comparisons, statistically significant ΔVEGF (*p* < 0.0007, SMD = −0.09), ΔPWA SP (*p* < 0.0057, SMD = −0.91), ΔPWV (*p* < 0.0189, SMD = −0.55), ΔTNF (*p* < 0.0471, SMD = −0.68) were observed (Table 3).

The proliferation grade of HUVEC exposed for 24 h to serum derived from the HD, LD, and placebo groups did not change significantly (Table 4).

In individuals who received probiotics after 12 weeks of study, a significant relationship was documented between the post-intervention SBP and the PWV values (*r* = 0.46), the post-intervention SBP PWA PP values (*r* = 0.54), and between the post-intervention DPB value and the ΔPWV (*r* = −0.43), and between the DBP value and the post-intervention TNF value (*r* = 0.49). The correlations between the IL-6 value and the PWA SP value after intervention (*r* = 0.46), the IL-6 value after intervention, and the ΔTNF (*r* = −0.48) and ΔTM (*r* = −0.39) values were documented. The post-intervention TM value was significantly associated with the post-intervention SBP value (*r* = 0.41), the post-intervention DBP value (*r* = 0.63), and the post-intervention PWA Alx value (*r* = −0.41) (Table 5).

## 4. Discussion

The previous literature has widely discussed the role of probiotics in the treatment of cardiovascular diseases. However, despite the research carried out, knowledge about this topic is still incomplete. In our work, we investigated the extent to which supplementation with selected multi-probiotics can improve the function of the arterial endothelium in obese individuals.

The novelty of this work is first, in the assessment of not only biochemical parameters, but also in the functional parameters indicating endothelial function, and, second, in the participation of postmenopausal obese women in the trial, who have a significantly increased risk of developing CVD. The third novel approach was the use of dose-dependent supplementation of a composed multispecies probiotic, Ecologic^®^ Barrier, containing a selected range of bacterial strains.

In recent years, the emphasis has been placed on the role of arterial stiffness in the development of cardiovascular diseases [32,33]. There is a multitude of available methods available to test this parameter, among which PWV is generally considered to be the most precise way to non-invasively estimate arterial stiffness in humans. Indeed, a wide range of studies has shown that high PWV values are associated with increased arterial stiffness and increased risk for cardiovascular disease [34,35]. PWA is another modality that evaluates arterial function [36]. The central aortic hemodynamic parameters derived by PWA include PP, AP, and Alx, reliable markers for estimating arterial stiffness [36]. A significant association between blood pressure and the PWV and PWA values was also present in our study. It should be noted that in our work, the initial PWV values did not cross the reference values (12 m/s), but we also rated this parameter in the studied groups after the intervention. When comparing PWV at the beginning of the study and after 12 weeks, a statistically significant decrease in the HD group was demonstrated. No similar trend was found in the LD and placebo groups. Interestingly, a significant decrease was observed in the PWV change in the HD group and LD group compared to the placebo after the intervention, as well as a difference depending on the dose of the probiotic. The way in which probiotics affect the endothelium is not fully understood and depends on a number of factors: the number of individual strains, the age of the study group, concomitant diseases, the method of administration, and the dosage of the preparation [22,37]. Menni et al. have shown that gut microbial diversity is associated with lower arterial stiffness in healthy women [38]. Other factors that may be involved in the development of arterial stiffness are insulin resistance and lipid metabolism disturbances [39]. In our previous study, we showed that the Ecologic^®^ Barrier supplementation favorably affects cardiovascular risk factors in a dose-dependent manner, showing beneficial effects on cardiometabolic parameters, including the lipid metabolism and insulin resistance, as well as gut permeability [25]. Sabico et al. observed that Ecologic^®^ Barrier supplementation for six months caused a significant decrease in insulin resistance, and improved the lipid and carbohydrate metabolism [24]. The drop in PWV in the studied population after the Ecologic^®^ Barrier supplementation seems to be the result of the above-mentioned phenomena, and this requires further research.

In the HD and LD groups, a significant decrease in SBP was observed between the values at the beginning and end of the study. The significant correlation between SBP and PWV demonstrated in our study confirms the dependence of PWV on the SBP value [40].

In terms of pre versus post-intervention values, similar trends were observed for PWA SP, PWA PP, and PWA Alx. After 12 weeks of intervention, significant differences were demonstrated for PWA SP and PWA Alx, and an effect depending on the dose was observed for PWA SP only. The obtained results are highly innovative, and so far, the above parameters have not been assessed in obese women after menopause undergoing a 12-week supplementation with the selected probiotic preparation.

An important issue is whether the decrease in PWV and PWA decrease due to probiotic supplementation improves endothelial function. To confirm this, it is necessary to find other parameters that are sensitive enough to reflect an improvement in endothelial function. However, current methods for estimating endothelial cell biomarkers show discrepancies. Most of the previous research, while reflecting endothelial cell dysfunction, displayed a decline in proinflammatory biomarkers [41,42]. In our study, PWA SP was significantly correlated with serum Il-6 concentration, and an inverse relation between IL-6 and delta TNF was documented. However, it should be emphasized that dysfunctions in smooth muscle cells may also contribute to an increased vascular tone, remodeling, and vasoreactivity in patients with cardiovascular diseases [43]. Undoubtedly, an extension of the research methods, e.g., the measurement of flow-mediated dilation or venous occlusion plethysmography, nitric oxide bioavailability, and reactive oxygen species level, would provide more information on the endothelium-mediated functional improvement of the patient after probiotic treatment. However, it is known that chronic inflammation is an important factor in the pathogenesis of endothelial dysfunction in obese people [44,45]. Disorders of the intestinal microbiota lead to the leakage of the intestinal barrier, which leads to the penetration of endotoxins into the blood and the subsequent development of the subclinical inflammatory process. The blood concentration of inflammatory cytokines such as TNF-α and Il-6 increases [46,47]. Accordingly, we observed significant decreases in TNF-α and Il-6 in probiotic-supplemented women, but not in the placebo group. In addition, in the case of TNF, the effect was dose-dependent. The ability to modulate inflammation depends on the type and strain of bacteria that are components of the probiotic preparation. Jafarneid et al. showed that a preparation containing the lactic acid bacteria reduced the concentrations of TNF-α and IL-6 in the group of women with gestational diabetes [48]. Similarly, the administration of *Lactobacillus casei* 01 bacteria to patients with rheumatoid arthritis significantly decreased TNF-α [49,50]. Our previous study, as well as a study by Subico et al., confirmed the anti-inflammatory- and intestinal barrier-improving properties of Ecologic^®^ Barrier [24,25].

Visceral fat is well vascularized, and it shows greater angiogenesis capacity; moreover, it more easily induces inflammation [51,52]. The role of TNF-α in the process of angiogenesis remains unclear. Perhaps TNF-α stimulates endothelial cells to increase the synthesis of proangiogenic factors such as interleukin-8 (Il-8), VEGF, and the basic fibroblast growth factor bFGF. The expression of Il-8 and VEGF is regulated by the activation of the nuclear transcription factor kappa Β (NF-κB), while bFGF is partially regulated by the activation of the AP-1 transcription factor [53]. The chronic effects of pro-inflammatory TNF-α on endothelial cells reduce the synthesis of VEGF and its receptors [54]. TNF-α is thought to be the major cytokine contributing to endothelial dysfunction, leading to inflammatory activation [55].

The administration of multispecies probiotic supplements significantly decreased the VEGF level. Previous investigations of the influence of probiotics on the VEGF level have been limited to a few mentioned studies, mainly in animal models, and in patients with non-obesity chronic inflammatory diseases. Marlicz et al. showed that short-term probiotic mixture (VSL#3) administration affects several clinical and biochemical parameters, including VEGF, which is commonly altered in liver cirrhosis [56]. Li et al. demonstrated that *Lactobacillus rhamnosus* reduces Lipopolysaccharides (LPS)-induced anti-inflammatory cytokines IL-1RN, IL-4 and IL-10, and LPS-induced VEGF output [57]. Other studies indicated that the probiotic yeast *Saccharomyces boulardii* can modulate angiogenesis to limit intestinal inflammation and to promote mucosal tissue repair by regulating VEGFR signaling [58]. It is well known that VEGF acts on endothelial cells by regulating its proliferation and migration grade. Therefore, we performed an in vitro study to assess the putative ability of serum derived from our patients before and after probiotic supplementation, to act on HUVEC proliferation. Surprisingly, despite the significantly decreased VEGF level observed in obese women supplemented with high doses of probiotics, both the mean changes in HUVEC number and the percentage of change of cells exposed to serum from the HD group showed increasing trends, although the differences were not quite significant. The discrepancy between the results may be explained by the antiangiogenic potential of VEGF. Moreover, experiments that measure the change of endothelial nitric oxide synthase expression and activity, reactive oxygen species production, tight junction protein level, and endothelial permeability in response to patient serum would provide a more mechanistic insight into the role of the endothelium. Recently, Ngo et al. demonstrated that impaired angiogenesis, which is associated with obesity, may be driven by the antiangiogenic isoform VEGF_165b_ [59]. The authors demonstrated that obese patients are characterized by elevated serum levels of VEGF_165_b compared to lean individuals. Recently, in an in vitro study, Yanagihara et al. proved that exposure to the probiotic *Lactobacillus acidophilus* L-92 modulates the expression of genes that are involved in splicing in epithelial Caco-2 cells [60]. The antibody used in our study does not distinguish between angiogenic and antiangiogenic VEGF; nevertheless, here, we demonstrated, for the first time, that multispecies probiotic supplementation could decrease VEGF serum levels in postmenopausal women with obesity in a dose-dependent manner; thus, studies including the usage of a VEGF_165b_ antibody are needed to draw further conclusions.

An important role of the endothelium is its involvement in the regulation of blood coagulation. Data concerning changes in other parameters of endothelial cell function, including as a mediator of coagulation and fibrinolysis, are limited in the literature. Our study demonstrated that a multispecies probiotic has a beneficial impact on the TM level in obese postmenopausal women by the significant decrease in the HD group observed after probiotic supplementation without any changes seen in the placebo-receiving individuals. TM is one of the most sensitive and specific markers of endothelial damage; it changes the properties of thrombin with a simple anticoagulant [61]. The concentration of free TM has been repeatedly found in high-risk CVD patients. Disconnected from the endothelium, transmembrane fragments can be considered as a marker of endothelial cell injury and thus, an early marker of atherosclerosis [61]. In our study, TM was significantly correlated with functional parameters of endothelium dysfunction, such as PWA Alx, or PWA AP, and blood pressure values. Concerning biochemical parameters, delta TM is inversely correlated with Il-6. The influence of targeted probiotics on TM in obesity has not been studied so far. Recently, Zelaya et al. studied the effects of intranasal *Lactobacillus Rhamnosus* therapy on coagulation parameters, including TM [62]. Our work is clinically relevant, and thus, for the first time, it demonstrates the ability of a specific probiotic supplementation to beneficially modulate a coagulation biomarker in obese postmenopausal women.

The strengths of the current study are the design (a randomized, placebo-controlled, double-blind intervention), the use of a panel of analyzed endothelial parameters (functional and biochemical), and the dose-dependent efficacy analysis.

The major limitation of this study is the relatively small number of individuals examined. The main reason for this was the rigorous inclusion and exclusion criteria. However, the applied criteria enabled us to select a homogenous group of subjects who were not affected by diseases or states that might have significantly influenced the results of the study. Although in the current, as well as our former study, we observed that probiotic administration resulted in an anti-inflammatory effect (decreased IL-6 and TNF-α), as well as a reduced LPS translocation, we consider the lack of the analysis of microbial composition and their metabolites in feces as another limitation which could demonstrate the influence of probiotic bacteria on the gut microbiota composition, as well as its potential metabolic activity. This missing information would shed more light on the mechanism of the probiotic effect on vascular function and arterial stiffness. It would be also interesting to perform more detailed studies of probiotics effects on the gut barrier.

## 5. Conclusions

In this randomized double-blind placebo-controlled 12-week trial, we showed, for the first time, that supplementation with the multispecies probiotic supplement Ecologic^®^ Barrier favorably modifies both functional and biochemical markers of vascular dysfunction in obese postmenopausal women. The role of multispecies probiotic supplements in cardiovascular prevention needs further investigation, but it appears that it could be a useful therapy for obese patients.

## Figures and Tables

**Table 1:** The baseline characteristics of studied parameters in the high dose (HD), low dose (LD), and placebo groups.

Variable	Group	Mean ± SD	SMD	*p*-Value	*p*-Value Post-Hoc Test
BMI (kg/m^2^)	HD	36.57 ± 5.95	0.09 *		ns
	LD	36.00 ± 5.20	−0.02 ^†^	0.9365	
	Placebo	36.10 ± 11.93	0.10 ^#^		
Age (years)	HD	55.16 ± 6.87	−0.50 *		ns
	LD	56.38 ± 6.55	−0.35 ^†^	0.2977	
	Placebo	58.72 ± 7.25	−0.18 ^#^		
Functional parameters					
SBP (mm Hg)	HD	134.80 ± 10.10	0.10 *		ns
	LD	133.50 ± 10.86	−0.01 ^†^	0.7391	
	Placebo	133.64 ± 12.20	0.12 ^#^		
DBP (mm Hg)	HD	79.88 ± 8.05	−0.51 *		ns
	LD	82.46 ± 5.53	−0.20 ^†^	0.1446	
	Placebo	83.76 ± 7.26	−0.37 ^#^		
PWA SP (mm Hg)	HD	131.32 ± 7.50	0.40 *		ns
	LD	126.56 ± 12.81	−0.05 ^†^	0.1964	
	Placebo	127.23 ± 12.43	0.45 ^#^		
PWA PP (mm Hg)	HD	46.44 ± 11.18	0.01 *		ns
	LD	41.88 ± 10.93	−0.41 ^†^	0.1515	
	Placebo	46.35 ± 10.65	0.41 ^#^		
PWA Alx	HD	33.28 ± 13.30	0.06 *		ns
	LD	32.36 ± 11.19	−0.02 ^†^	0.8618	
	Placebo	33.62 ± 10.30	0.08 ^#^		
PWV (m/s)	HD	7.32 ± 0.90	0.46 *		ns
	LD	6.92 ± 1.78	0.01 ^†^	0.2248	
	Placebo	6.90 ± 0.94	0.28 ^#^		
Biochemical parameters					
IL-6 (pg/mL)	HD	4.50 ± 0.51	0.08 *		ns
	LD	4.71 ± 0.49	0.48 ^†^	0.1887	
	Placebo	4.46 ± 0.56	−0.42 ^#^		
VEGF (pg/mL)	HD	155.04 ± 19.38	0.81 *		* 0.0192
	LD	142.46 ± 27.24	0.19 ^†^	0.0034	
	Placebo	137.64 ± 23.20	0.53 ^#^		
TNF (pg/L)	HD	1.04 ± 0.34	−0.03 *		ns
	LD	1.24 ± 0.36	0.54 ^†^	0.0663	
	Placebo	1.05 ± 0.34	−0.57 ^#^		
TM (ng/mL)	HD	4.20 ± 0.73	0.02 *		ns
	LD	4.01 ± 0.58	−0.30 ^†^	0.5170	
	Placebo	4.19 ± 0.64	0.29 ^#^		
vWF (ng/mL)	HD	84.86 ± 6.40	0.11 *		ns
	LD	83.90 ± 5.71	−0.05 ^†^	0.8522	
	Placebo	84.18 ± 6.47	0.16 ^#^		

Data are the arithmetic mean ± SD; SMD: standardized mean difference; ns: not significant; BMI: body mass index; DBP: diastolic blood pressure; Il-6: interleukin-6; PWA Alx: pulse wave analysis augmentation index; PWA PP: pulse wave analysis pulse pressure; PWA SP: pulse wave analysis systolic pressure; PWV: pulse wave velocity; SBP: systolic blood pressure; TM: thrombomodulin; TNF: tumor necrosis factor alpha; VEGF: vascular endothelial growth factor; vWF: von Willebrand factor. * HD: high dose of the probiotic group vs. placebo; ^†^ LD: low dose of the probiotic group vs. placebo; ^#^ HD vs. LD.

**Table 2:** The comparison of the tested parameters in the high dose (HD), low dose (LD), and placebo groups at the beginning of the study and after three months of supplementation.

Variable	Group	Baseline	After 3 Months	SMD	*p*-Value
BMI	HD	36.57 ± 5.95	36.22 ± 5.29	0.31	0.3165
	LD	36.00 ± 5.20	35.51 ± 5.16	0.39	0.1209
	Placebo	36.10 ± 4.37	36.04 ± 4.32	0.07	0.9612
Functional parameters					
SBP (mm Hg)	HD	134.80 ± 10.10	131.40 ± 9.41	0.39	**0.0359**
	LD	133.50 ± 10.86	130.88 ± 9.42	0.26	**0.0498**
	Placebo	133.64 ± 12.20	131.52 ± 12.31	0.17	0.1795
DBP (mm Hg)	HD	79.88 ± 8.05	79.36 ± 7.42	0.07	0.7031
	LD	82.46 ± 5.53	81.73 ± 6.40	0.12	0.5095
	Placebo	83.76 ± 7.26	81.88 ± 7.20	0.26	0.2927
PWA SP (mm Hg)	HD	131.32 ± 7.50	121.75 ± 11.14	1.01	**0.0004**
	LD	126.56 ± 12.81	127.28 ± 16.53	−0.05	0.6997
	Placebo	127.23 ± 12.43	127.92 ± 16.79	−0.04	0.8078
PWA PP (mm Hg)	HD	46.44 ± 11.18	40.63 ± 8.65	0.67	**0.0103**
	LD	41.88 ± 10.93	41.45 ± 14.07	0.03	0.7989
	Placebo	46.35 ± 10.65	44.16 ± 8.50	0.26	0.3219
PWA Alx	HD	33.28 ± 13.30	27.56 ± 9.94	0.58	**0.0021**
	LD	32.36 ± 11.19	33.48 ± 11.12	−0.10	0.1711
	Placebo	33.62 ± 10.30	33.19 ± 11.41	0.04	0.8751
PWV (m/s)	HD	7.32 ± 0.90	6.45 ± 0.77	1.13	**0.0013**
	LD	6.92 ± 1.78	6.74 ± 1.29	0.14	0.3536
	Placebo	6.90 ± 0.94	6.79 ± 0.95	0.12	0.3221
Biochemical parameters					
IL-6 (pg/mL)	HD	4.50 ± 0.51	4.41 ± 0.59	0.15	**0.0173**
	LD	4.71 ± 0.49	4.50 ± 1.15	0.43	**0.0396**
	Placebo	4.46 ± 0.56	4.45 ± 4.45	0.02	0.3282
VEGF (pg/mL)	HD	155.04 ± 19.38	121.52 ± 32.66	1.73	**0.0004**
	LD	142.46 ± 27.24	147.35 ± 34.19	−0.18	0.5991
	Placebo	137.64 ± 23.20	137.32 ± 29.94	0.01	0.7467
TNF (pg/mL)	HD	1.04 ± 0.34	0.85 ± 0.23	0.56	**0.0001**
	LD	1.24 ± 0.36	1.21 ± 052	0.08	0.2427
	Placebo	1.05 ± 0.34	1.04 ± 0.31	0.03	0.7610
TM (ng/mL)	HD	4.20 ± 0.73	3.77 ± 0.66	0.59	**0.0006**
	LD	4.01 ± 0.58	4.03 ± 0.59	−0.03	0.8081
	Placebo	4.19 ± 0.64	4.28 ± 0.74	−0.14	0.5938
vWF (ng/mL)	HD	84.86 ± 6.40	84.80 ± 6.45	0.01	0.8929
	LD	83.90 ± 5.71	84.16 ± 5.58	−0.05	0.8081
	Placebo	84.18 ± 6.47	83.55 ± 5.96	0.10	0.9544

Significant differences are highlighted in bold. Data are the arithmetic mean ± SD; SMD: standardized mean difference; BMI: body mass index; DBP: diastolic blood pressure; Il-6: interleukin-6; PWA Alx: pulse wave analysis augmentation index; PWA PP: pulse wave analysis pulse pressure; PWA SP: pulse wave analysis systolic pressure; PWV: pulse wave velocity; SBP: systolic blood pressure; TM: thrombomodulin; TNF: tumor necrosis factor alpha; VEGF: vascular endothelial growth factor; vWF: von Willebrand factor.

**Table 3:** The changes in the biochemical and functional variables in the high dose (HD), low dose (LD), and placebo groups after three months.

Variable	Group	Mean ± SD	SMD	*p*-Value	*p*-Value Post-Hoc Test
ΔBMI	HD	−0.35 ± 1.29	−0.26 *		ns
	LD	−0.49 ± 1.29	−0.38 ^†^	0.6960	
	Placebo	−0.06 ± 0.87	0.11 ^#^		
Functional parameters					
ΔSBP (mm Hg)	HD	−3.76 ± 8.47	−0.21 *		ns
	LD	−2.62 ± 9.63	−0.06 ^†^	0.7789	
	Placebo	−2.12 ± 6.83	−0.13 ^#^		
ΔDBP (mm Hg)	HD	−0.52 ± 6.74	0.17 *		ns
	LD	−0.73 ± 5.57	0.16 ^†^	0.7677	
	Placebo	−1.88 ± 8.74	0.03 ^#^		
Δ PWA SP (mm Hg)	HD	−9.57 ± 10.87	−1.00 *		* **0.0054** ^#^ **0.0057**
	LD	0.72 ± 12.49	−0.01 ^†^	**0.0016**	
	Placebo	0.69 ± 10.61	−0.91 ^#^		
ΔPWA PP (mm Hg)	HD	−5.92 ± 10.55	−0.37 *		
	LD	−1.70 ± 9.97	0.02 ^†^	0.1439	ns
	Placebo	−1.88 ± 11.10	−0.41 ^#^		
ΔPWA Alx	HD	−5.72 ± 8.84	−0.55 *		* **0.0079**
	LD	1.12 ± 4.73	0.19 ^†^	**0.0084**	
	Placebo	−0.43 ± 10.45	−0.97 ^#^		
ΔPWV (m/s)	HD	−0.87 ± 1.69	−0.82 *		* **0.0045** ^#^ **0.0189**
	LD	−0.19 ± 1.76	−0.07 ^†^	**0.0028**	
	Placebo	−0.10 ± 0.58	−0.55 ^#^		
Biochemical parameters					
ΔIL-6 (pg/mL)	HD	−0.09 ± 0.02	−0.38 *		ns
	LD	−0.21 ± 0.99	−0.10 ^†^	0.2461	
	Placebo	−0.009 ± 0.08	−0.09 ^#^		
ΔVEGF (pg/mL)	HD	−33.52 ± 36.84	−1.09 *		* **0.0001** ^#^ **0.0007**
	LD	4.88 ± 33.08	0.20 ^†^	**0.0001**	
	Placebo	−0.58 ± 21.75	−1.10 ^#^		
ΔTNF (pg/mL)	HD	−0.20 ± 0.22	−1.03 *		* **0.0009** ^#^ **0.0471**
	LD	−0.03 ± 0.28	−0.09 ^†^	**0.0012**	
	Placebo	−0.01 ± 0.14	−0.68 ^#^		
ΔTM (ng/mL)	HD	−0.43 ± 0.52	−0.78 *		* **0.0194**
	LD	−0.14 ± 1.03	−0.25 ^†^	**0.0172**	
	Placebo	0.09 ± 0.79	−0.36 ^#^		
ΔvwF (ng/mL)	HD	−0.07 ± 6.64	0.07 *		ns
	LD	0.12 ± 5.13	0.11 ^†^	0.9915	
	Placebo	−0.44 ± 4.64	−0.03 ^#^		

Significant differences are highlighted in bold. Data are the arithmetic mean ± SD; SMD: standardized mean difference; ns: not significant; BMI: body mass index; DBP: diastolic blood pressure; Il-6: interleukin-6; PWA Alx: pulse wave analysis augmentation index; PWA PP: pulse wave analysis pulse pressure; PWA SP: pulse wave analysis systolic pressure; PWV: pulse wave velocity; SBP: systolic blood pressure; TM: thrombomodulin; TNF: tumor necrosis factor alpha; VEGF: vascular endothelial growth factor; vWF: von Willebrand factor; Δ: change of the parameter. * HD: high dose of the probiotic group vs. Placebo; ^†^ LD: low dose of the probiotic group vs. Placebo; ^#^ HD vs. LD.

**Table 4:** The proliferation grade of human umbilical vein endothelial cells (HUVEC) exposed for 24 h to serum derived from the high dose (HD), low dose (LD), and placebo groups before and after the intervention, estimated by an in vitro MTT colorimetric assay.

Variable	Study Population			
	Group	Mean ± SD	SMD	*p*-Value
% of control before	HD	144.03 ± 43.18	−0.67 *	0.1042
	LD	160.83 ± 32.07	−0.30 ^†^	
	Placebo	171.23 ± 37.51	−0.44 ^#^	
% of control after	HD	159.68 ± 38.42	0.22 *	0.4502
	LD	163.50 ± 33.88	0.33 ^†^	
	Placebo	151.12 ± 39.96	−0.11 ^#^	
Mean Δ	HD	15.65 ± 54.28	0.72 *	0.0529
	LD	2.67 ± 30.78	0.60 ^†^	
	Placebo	−20.11 ± 44.17	0.29 ^#^	
% of change	HD	27.94 ± 77.65	0.65 *	0.0513
	LD	3.28 ± 20.48	0.57 ^†^	
	Placebo	−9.77 ± 25.29	0.43 ^#^	
% of control before vs. after	HD	144.03 vs. 159.68	−0.27	0.1821
	LD	160.83 vs. 163.50	−0.09	0.7716
	Placebo	171.23 vs. 151.12	0.44	0.0520

Data are the arithmetic mean ± SD; SMD: standardized mean difference; Δ: change of the parameter. Control; means cells maintained in standard culture medium without patient serum. * HD: high dose of the probiotic group vs. Placebo; ^†^ LD: low dose of the probiotic group vs. Placebo; ^#^ HD vs. LD.

**Table 5:** The significant correlations in the high dose (HD) and low dose (LD) groups.

Group	Correlated Parameters	*p*	*r*
**LD**	SBP II & PWV II	0.0181	0.46
	DBP II & Δ PWV	0.0252	−0.43
	IL-6 II & Δ TM	0.0482	−0.39
	sTM II & PWA Alx II	0.0401	−0.41
**HD**	SBP II & PWA PP II	0.0174	0.54
	DBP II & TNF II	0.0121	0.49
	IL-6 II & PWA SP II	0.0229	0.46
	IL-6 II & Δ TNF	0.0249	−0.48
	TM II & SBP II	0.0278	0.41
	TM II & DBP II	0.0058	0.63

I: the value of the parameter at baseline; II: the value of the parameter after completion; Δ: change of the parameter; *r*: Spearman correlation index. DBP: diastolic blood pressure; Il-6: interleukin-6; PWA Alx: pulse wave analysis augmentation index; PWA PP: pulse wave analysis pulse pressure; PWA SP: pulse wave analysis systolic pressure; PWV: pulse wave velocity; SBP: systolic blood pressure; TM: thrombomodulin; TNF: tumor necrosis factor alpha.
